# Ageing, multimorbidity, and quality of life: a mediation analysis using longitudinal ageing study in India

**DOI:** 10.3389/fpubh.2025.1562479

**Published:** 2025-04-25

**Authors:** Bharti Singh, Ajay Kumar

**Affiliations:** ^1^Department of Survey Research and Data Analytics, International Institute for Population Sciences (IIPS), Mumbai, India; ^2^Department of Biostatistics and Epidemiology, International Institute for Population Sciences (IIPS), Mumbai, India

**Keywords:** multimorbidity, quality of life, India, ageing, structural equation model

## Abstract

**Background:**

The ageing population in India is growing rapidly, but the decline in healthy life expectancy is more pronounced. This trend has been compounded and constituted by poor quality of life (QoL), with the salient underlying role of multimorbidity as the leading risk factor. This study primarily aimed to assess the intermediating role of multimorbidity as the risk factor for exogenous socioeconomic and demographic factors on QoL.

**Methods:**

This study used data from 73,396 individuals aged 45 years and above from the Longitudinal Ageing Study in India (LASI), Wave – 1, 2017–18. Multimorbidity was defined as the simultaneous existence of two or more chronic conditions in an individual. The QoL score was constructed using Principal Component Analysis (PCA) by utilizing 21 factors under six domains (physical health, psychological health, social relationship, environmental satisfaction, life satisfaction and general health), with the composite QoL score ranging from 0 to 100. Further, the Structural equation model (SEM) was used to assess the role of multimorbidity as the intermediating risk factor for exogenous factors on QoL.

**Results:**

Distributions of morbidities burden were skewed toward non-communicable diseases (NCDs) and sequentially escalated multimorbidity burden was observed among the oldest of old age groups. After the age of 75, there was a steep decline in the gradient of QoL score. The SEM results showed a substantial rise in multimorbidity burden leading to poor QoL with a magnitude of *β* = −2.39, *p* < 0.001. Age and sex of the respondents exhibited a significant negative impact on QoL, impacting it directly (*β* = −1.25; *β* = −1.19) as well as indirectly through multimorbidity (*β* = −0.11). In contrast, childhood health demonstrated a solely direct impact on QoL, with no significant indirect pathway through multimorbidity. This study further revealed that urban residence had a pronounced positive direct effect on QoL (*β* = 0.9, *p* < 0.001).

**Conclusion:**

This study underscores the role of multimorbidity as a key mediator between socioeconomic and demographic factors on QoL among older adults in India. With the increasing prevalence of multimorbidity, policies should prioritize integrated geriatric healthcare services. Strengthening healthcare for early screening and affordable chronic disease management is essential.

## 1 Introduction

The coexistence of two or more chronic conditions or morbidity is known as multimorbidity, which has emerged as a global health concern in high-income countries (1, 2). In recent decades, this trend has been compounded by the rapidly ageing population and epidemiological transition toward non-communicable diseases (NCDs), particularly in low-and middle-income countries (LMICs), where the burden of multimorbidity has reached alarming levels (2–4). The World Health Organization’s (WHO) Study on Global Ageing and Adult Health (SAGE) Wave 1, conducted between 2007 and 2010, reports that the prevalence of morbidity and multimorbidity in the pooled sample of these six countries was 54.2% in Russia and 21.9% in China, where the rural population (22.9%) exhibits a higher prevalence of multimorbidity than the urban population (20.5%), and females (24.8%) demonstrate a higher rate of multimorbidity than males (19.0%) (5).

The burden of multimorbidity consistently increases in LMIC, wherein a significant proportion of the older adult population suffers from multimorbidity, due to health system challenges, socioeconomic disparities, and a combination of higher infectious diseases, NCDs with a declining rate of mortality (3, 6–10). This phenomenon is particularly evident in India, which is currently undergoing through a rapid demographic shift and shares a significant ageing population burden. This surge of multimorbidity among India’s ageing population underscores the urgent need to align healthcare strategies with the Sustainable Development Goals (SDGs). Multimorbidity amplifies inequalities, directly challenging SDG 3 (Good Health and Wellbeing) and SDG 10 (Reduced Inequalities). As SDG 3.4 targets reducing premature mortality from non-communicable diseases (NCDs) and SDG 3.8 emphasizes universal health coverage, addressing multimorbidity becomes crucial for achieving these targets, globally.

According to the Indian Ageing Report 2023, 23 % of Indians suffer from multimorbidity, where states like Nagaland had the lowest prevalence of multimorbidity (7.3%) and Kerla had the highest burden of multimorbidity (52.2%) (11). Nonetheless, several prior studies have explored various socioeconomic, demographic, and ecological key drivers of multimorbidity burden (3, 4, 12). A case study conducted in 2015 in West Bengal and Odisha revealed that females who had a higher risk of multimorbidity were more susceptible to multimorbidity than males (7). Furthermore, low income, poor education level, and limited access to healthcare are associated with increased multimorbidity as well as diminished Quality of life (QoL) (13, 14). A recent study done by Dolui et al. (2023) revealed that in India, the population belonging to a higher socioeconomic background is more likely to suffer from multimorbidity (15). Further, Arthritis, cataract, and hypertension are the most common chronic conditions among Indians (16).

QoL can be defined as the extent to which an individual experiences satisfaction, comfort, wellbeing, and the capacity to engage in life events. According to WHO (17), QoL is an “individual’s perception of their position in life in the context of the culture and value system in which they lived and in relation to their goals, expectations, standards, and concerns.” This definition incorporates physical health, psychological state, level of independence, social relationships, personal beliefs, and their relationship with salient features of the environment. Therefore, the QoL is measured across multiple domains (18).

Several studies have also shown that multimorbidity alone can have a significant impact on the QoL of the ageing population (13, 19–21). Individuals with multimorbidity often lag in the physical, psychological, social, and functional aspects of life, which leads to reduced ability to perform daily activities and increased health costs (22). Furthermore, multimorbidity can take a toll on mental health, leading to depression, anxiety, and reduced psychological wellbeing (23, 24). The burden of managing multimorbidity along with emotional distress in dealing with chronic illnesses can further exacerbate mental health issues. Social wellbeing is also affected, as individuals with multimorbidity may face social isolation due to limitations in participation, stigma associated with illness, or changes in social roles and relationships (13, 25).

The complex relationship between multimorbidity and QoL in older adults is a critical area of study, particularly for designing effective interventions that enhance wellbeing. While the existing literature establishes the adverse impact of multimorbidity on QoL, the underlying pathways through which this relationship unfolds remain inadequately examined. Furthermore, most studies have focused on direct associations, overlooking the mediation effects that may operate through socioeconomic status, lifestyle behaviors, or psychological wellbeing. Additionally, the potential interaction effects where specific demographic or health-related factors may exacerbate or mitigate the influence of multimorbidity on QoL remain underexplored. Given India’s diverse socio-cultural and economic landscape, understanding these mechanisms is crucial for tailoring policies that address the heterogeneity in ageing experiences. Hence, to address this gap, our study aims to estimate the direct and indirect effects of socioeconomic, lifestyle, and demographic factors mediated by multimorbidity on QoL among older adults in India using nationally representative data from the Longitudinal Ageing Study in India (LASI), wave 1.

## 2 Materials and methods

### 2.1 Study population

This study used the national representative Longitudinal Ageing Study in India (LASI), Wave 1, 2017–18, survey dataset that provides exhaustive characteristics of the broad number of morbidities, for adults aged 45 years and above with the socioeconomic and demographic details, covering 28 states and 8 Union territories (UT) of India. LASI has adopted a multi-stage stratified areas probability cluster sampling design, with three stages in the rural and four stages in the urban areas, respectively. Firstly, a primary sampling unit (PSU) was selected from each state/union territory (UT), followed by a village (from rural) or ward (from urban) area in the second stage. Finally, households were selected from the rural areas. However, in urban areas, census enumeration blocks (CEB) were selected randomly from each urban area, after which households were chosen from the selected CEB (International Institute for Population Sciences, 2020). This study utilized the merged information of individual and biomarker datasets. The dataset contained samples of 73,396 adults aged 45 years and above, and in this research work, we primarily analysed data from 66,606 individuals after handling the missing data.

### 2.2 Defining morbidities and multimorbidity

For this study, we considered 14 chronic and severe morbidities using the information on all self-reported and diagnosis available in LASI, which were documented based on responses to the question, “Has any health professional ever diagnosed you with the following chronic conditions or diseases?” and “In the past 2 years, have you had any of the following diseases?.” These morbidities or chronic conditions include Musculoskeletal Disorders, Hypertension, Stroke, Heart Diseases, Chronic Lung Diseases, Neurological and Psychiatric disorders, Diabetes, Jaundice or Hepatitis, Tuberculosis, Cancer, Cholesterol, Hearing disorders, Gastrointestinal conditions, and Urogenital diseases. All morbidities were classified into binary form: absent or present. The morbidity score was generated and further categorized into two groups: No Multimorbidity (individuals who did not have two or more morbidities/chronic diseases) and Multimorbidity (individuals who had combinations of two or more morbidities/chronic diseases).

### 2.3 Quality of Life (QoL)

The World Health Organization (WHO) defines QoL as “an individual’s perception of their position in life in the context of the culture and value systems in which they live and about their goals, expectations, standards and concerns (17).” The WHO-QoL was based on a four-domain structure (physical health, psychological, social relationships, and environmental), representing an individual’s overall wellbeing and satisfaction with life, entirety. This definition reflects the perspective that QoL pertains to a subjective evaluation situated within a cultural, social, and environmental context.

This study used 21 factors under six domains, i.e., physical health, psychological health, social relationship, environmental satisfaction, life satisfaction and general health. A comprehensive composite index was constructed using Principal Component Analysis (PCA) to measure the QoL. To assess the adequacy of the sample for factor analysis, we conducted the Kaiser-Meyer-Olkin (KMO) Measure of Sampling Adequacy. The resulting KMO value was 0.80, indicating that the sample is well-suited for PCA. The PCA results identified six distinct domains that were retained based on the eigenvalue criteria. These six domains collectively accounted for 52% of the total variance, suggesting a reasonably strong explanatory power. Additionally, we assessed the construct validity using Cronbach’s alpha, which yielded a value of 0.69, demonstrating satisfactory internal consistency and acceptable construct validity. The QoL score was transformed into a linear scale, ranging from 0 to 100. A higher score indicated better QoL and vice versa. Detailed results of the PCA are provided in the Supplementary Tables S2–S5 and Supplementary Figure S1.

The survey used the Likert scale to measure different aspects of sub domains in all six domains. The physical health of an individual was assessed by considering the Activities of Daily Living (dressing, bathing, walking, eating, getting out of bed, using the toilet), physical energy and sleep comfort. Psychological wellbeing examined through self-reported inner peace, positive and negative feelings, satisfaction, spirituality, and concentration ability. The environmental aspects of QoL were assessed by financial status, feelings about safety, and satisfaction with living arrangements. The social domain was examined by living arrangements and the number of friends. Life satisfaction and general health were evaluated by individual questions. Two overall items measured general QoL, and the tool contained two culture-specific questions (i.e., “Do you feel respected by others?” and “Are you usually able to get the things you like to eat?”). All questions were recoded into dichotomous variables for further statistical analysis.

### 2.4 Exogenous factors

In this study, we considered the (45–49, 50–54, 55–59, 60–64, 65–69, 70–74, 75–79, 80+), Sex (Male/Female), BMI (Underweight, Normal, Overweight, Obese), Childhood health (Very good, Good, Fair, Poor, Very Poor), Working Status (Never worked, Currently working, Currently not working), Physical activity (Everyday, Weekly, Casual), Level of education (No schooling, < 5 Years, 5–9 years, 10 + years), MPCE quintiles (poorest, poorer, middle, richer, richest), Caste (SC/ST, OBC, Others), Religion (Hindu, Muslim, Christian and Others), and Residence (Rural, Urban). Further, detailed descriptions of all the exogenous factors are provided in the Supplementary Table S1.

### 2.5 Statistical analysis

#### 2.5.1 Prevalence

Age-specific prevalence rates (*P*) were calculated for morbidities and multimorbidity burdens among older adults in India, which are calculated as follows


$$P=\frac{\mathrm{Population}\;\mathrm{with}\;\mathrm{the}\;\mathrm{disease}\;\mathrm{or}\;\mathrm{condition}\;\mathrm{at}\;a\;\mathrm{specified}\;\mathrm{time}}{\mathrm{Population}\;\mathrm{at}\;\mathrm{risk}\;\mathrm{at}\;\mathrm{the}\;\mathrm{specified}\;\mathrm{time}}\times 100$$

#### 2.5.2 Structural equation model (SEM)

To understand the role of multimorbidity as the intermediating risk factor for exogenous factors on QoL, SEM was utilized with exogenous variables along with multimorbidity and endogenous variable (QoL). Path analysis was used to identify both the direct and indirect relationships in the model. In addition, standardized and unstandardized regression coefficients (*β*) and *p*-values with 95% CI were used to determine significant direct, indirect, and total effects.

The equation for exogenous variables:


$$\mathrm{Xi}=\lambda_{\mathrm{xi}}\xi +\delta_{i}$$

Where,

Xi are the observed variables related to an exogenous latent variable.

$\lambda_{\mathrm{xi}}$ are the factors loading for each observed variable on the latent construct.

ξ represent the exogenous latent variable.

$\delta_{i}$ represent the measurement error for each observed variable.

The equation for endogenous variables is:


$$Yj=\lambda_{yj}\eta +\varepsilon_{j}$$

Yj are the observed variables associated with the endogenous latent variables.

$\lambda_{yj}$ are the factors loading for each observed variable on the endogenous construct.

η represents the endogenous latent variables.

$\varepsilon_{j}$ represents the measurement errors for each observed variable.

SEM: equation of hypothesized causal relationships


η=Bη+Γξ+ζ

Where,

η is the vector of the endogenous latent variables.

B is a matrix of coefficients that represents the relationships among the endogenous latent variables.

Γ is the matrix that represents the effect of exogenous variables on the endogenous variables.

ξ represent exogenous latent variables.

ζ is the vector of error terms affecting the endogenous variables.

The SEM model fit was assessed using the following indices: ratio of $X^{2}$ to the respective degrees of freedom ( $X^{2}$ /df), goodness-of-fit (GIF) index, comparative fit index (CFI) and root mean square error of approximation (RMSEA). In addition, the Akaike Information Criterion (AIC) was applied to model selection; a smaller value AIC indicates a better model fit. All analyses were performed with R Studio version 2023.12.1 + 402,^1^ SAS and STATA 16.

## 3 Results

### 3.1 Prevalence of morbidities and multimorbidity at the national level

Table 1 presents that over half (51.4%) of older adults in India experienced multimorbidity. In the wide spectrum of disease burden, hypertension (26.7%), followed by gastrointestinal disorders (18%), and musculoskeletal disorders (16.2%), showed the highest prevalence, highlighting the high burden of acute and chronic morbidities. Severe morbidities with the highest mortality risk, such as, cancer (0.6%), tuberculosis (1.0%), and stroke (1.9%) had the lowest burden among the older adults in India.

**Table 1:** Multimorbidity and morbidity prevalence among older adults, LASI-Wave 1 (2017–18), India.

Causes	Weighted percentage *N* = 64,448 (95% CI)
Multimorbidity	51.4 (51.0, 51.8)
**Morbidities and conditions**	
Hypertension	26.7 (26.4, 27.1)
Gastrointestinal disorders	18.0 (17.7, 18.3)
Musculoskeletal disorder	16.2 (15.9, 16.5)
Diabetes	12.2 (11.9, 12.4)
Hearing disorder	6.9 (6.7, 7.1)
Lung diseases	6.6 (6.5, 6.8)
Urogenital diseases	6.5 (6.3, 6.6)
Heart diseases	3.8 (3.6, 3.9)
Jaundice or hepatitis	2.8 (2.7, 2.9)
Psychiatric or neurological disorder	2.4 (2.4, 2.6)
High cholesterol	2.2 (2.1, 2.3)
Stroke	1.9 (1.8, 2.0)
Tuberculosis	1.0 (0.1, 1.1)
Cancer	0.6 (0.6, 0.7)

**Figure 1:**
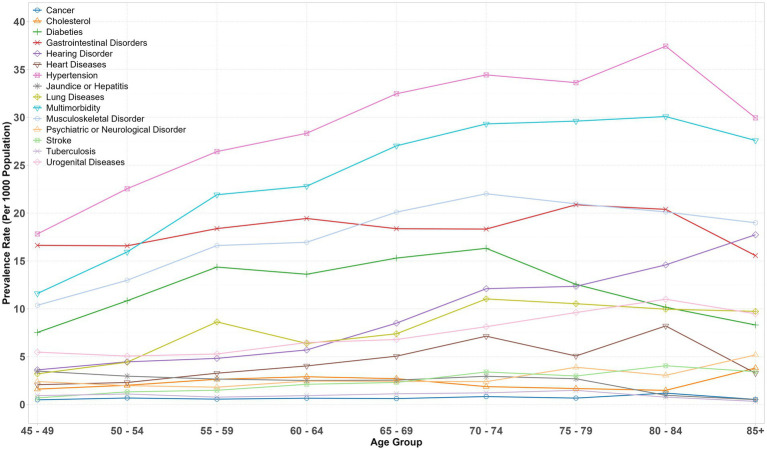
Age-specific weighted prevalence rates of morbidities and multimorbidity, LASI-Wave 1 (2017–18), India.

Figure 1 shows the prevalence pattern of morbidities and multimorbidity across the age groups. A strong association was observed in prevalence rates of morbidities and multimorbidity, with hypertension showing the steepest incline, followed by multimorbidity and musculoskeletal disorders. Other severe conditions, such as cancer and tuberculosis, exhibited the lowest prevalence rates across all age groups. Additionally, there was a consistent rise in the age-specific prevalence rates of hearing disorders across all age groups.

### 3.2 Characteristics of multimorbidity and QoL

**Figure 2:**
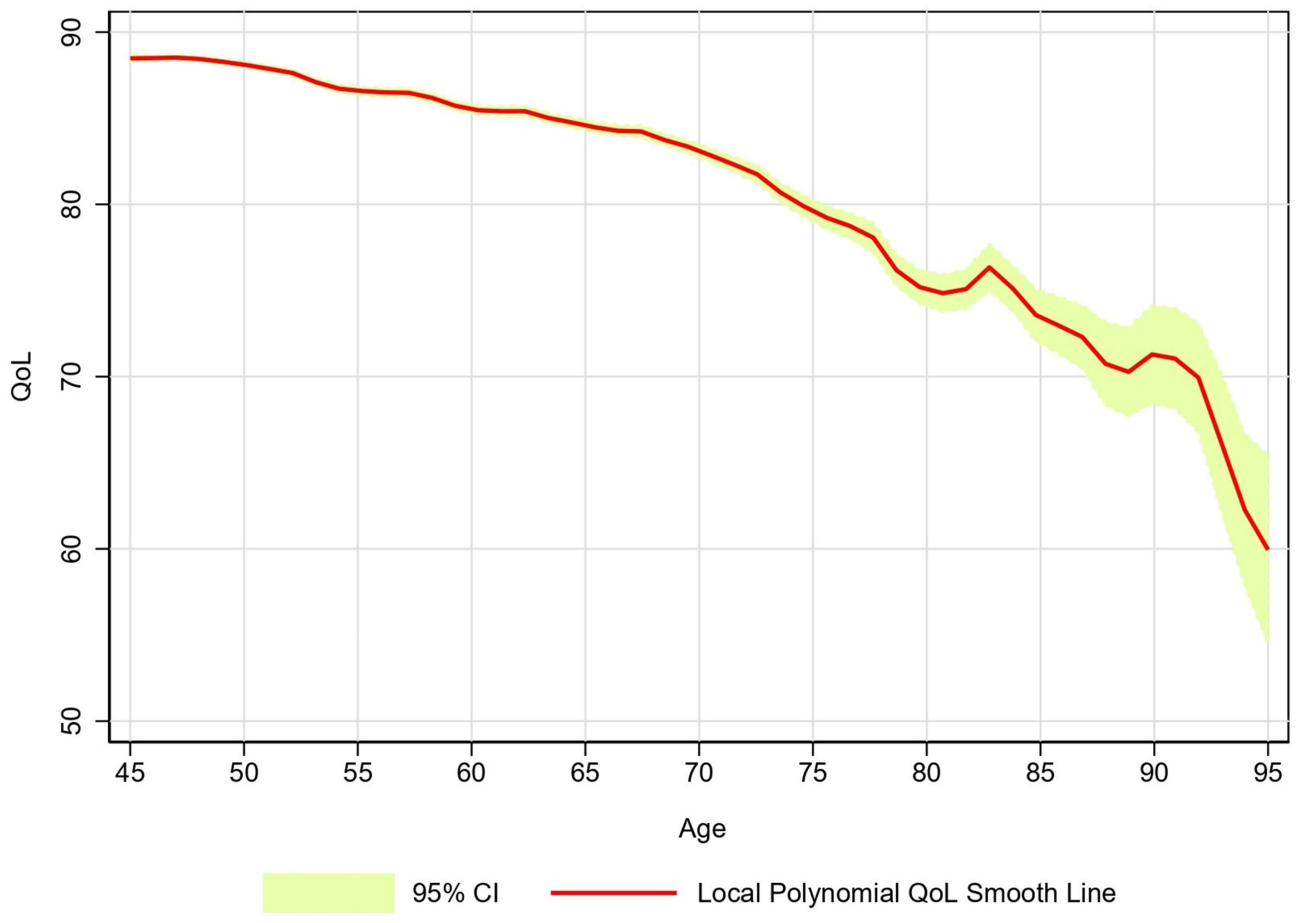
Age-specific smoothed QoL score, LASI-Wave 1 (2017–18), India.

Figure 2 depicts the local polynomial smoothed estimates of the QoL score over age. The graph revealed a decremental decrease in the QoL score with advancing age and a pronounced decline in the gradient was observed after 75 years of age.

Table 2 illustrates the mean QoL score and multimorbidity prevalence among exogenous factors. The prevalence of multimorbidity in the 45–49 age group was 39.4% and increased to 64% by the age of 85 and above, while the mean QoL score decreased from 88.6 to 71.3 in the same age groups. Females exhibited a higher prevalence of multimorbidity (51.8%) compared to males (49.7%). Additionally, their average quality of life (QoL) score was lower at 84.6, whereas males had a slightly higher mean QoL score of 86.8.

**Table 2:** Multimorbidity prevalence and mean QoL score by exogenous factors among older adults, LASI-Wave 1 (2017–18), India.

Exogenous factors	Multimorbidity (*N =* 64,448)		Mean QoL score (*N =* 64,448)	
	Percentage (95% CI)	Chi-square test	Mean Score	Kruskal Wallis test
Age group				
45–49	39.4 (38.6, 40.3)	*p* < 0.001	88.6	*p* < 0.001
50–54	45.0 (44.1, 45.8)		87.7	
55–59	50.4 (49.1, 51.6)		87.0	
60–64	51.7 (50.7, 52.7)		85.8	
65–69	57.6 (56.8, 58.9)		84.6	
70–74	59.8 (58.6, 61.1)		82.4	
75–79	64.8 (63.4, 66.2)		79.0	
80–84	62.7 (59.7, 65.6)		77.0	
85 +	64.0 (61.3, 66.5)		71.4	
Sex				
Male	49.7 (49.1, 50.3)	*p* < 0.001	86.8	*p* < 0.001
Female	51.8 (51.2, 52.3)		84.6	
BMI^b^				
Underweight	39.5 (38.7, 40.4)	*p* < 0.001	84.4	*p* < 0.001
Normal	47.9 (47.3, 48.4)		86.4	
Overweight	61.7 (60.9, 62.6)		86.7	
Obese	71.2 (69.8, 72.6)		85.2	
Childhood health				
Poor	57.3 (54.2, 60.4)	*p* < 0.001	80.1	*p* < 0.001
Moderate	49.9 (48.9, 51.0)		84.1	
Good	50.9 (50.4, 51.3)		85.9	
Physical activity				
Everyday	42.8 (42.0, 43.5)	*p* < 0.001	88.2	*p* < 0.001
Weekly	42.4 (41.2, 43.6)		87.2	
Casual	55.3 (54.8, 55.8)		84.4	
Working status				
Never worked	55.7 (55.0, 56.5)	*p* < 0.001	89.1	*p* < 0.001
Currently working	43.7 (43.2, 44.3)		85.7	
Currently not working	60.5 (59.8, 61.3)		76.8	
Highest level of schooling				
No Schooling	44.1 (43.6, 44.7)	*p* < 0.001	84.1	*p* < 0.001
< 5 Years	55.6 (54.5, 56.8)		85.9	
5–9 Years	55.7 (54.8, 56.5)		86.6	
10 + Years	61.2 (60.3, 62.1)		88.5	
MPCE quintile^a^				
Poorest	39.8 (38.9, 40.6)	*p* < 0.001	85.0	*p* < 0.001
Poorer	48.4 (47.5, 49.2)		85.7	
Middle	50.2 (49.3, 51.4)		85.7	
Richer	55.3 (54.4, 56.2)		85.9	
Richest	62.6 (61.7, 63.5)		85.8	
Caste category				
ST	47.9 (47.0, 48.8)	*p* < 0.001	85.1	*p* < 0.001
SC	30.9 (29.6, 32.0)		86.6	
OBC	50.4 (49.8, 51.0)		85.7	
Other	59.9 (59.1, 60.7)		85.4	
Religion				
Hindu	49.5 (49.1, 49.9)	*p* < 0.001	85.5	*p* < 0.001
Muslim	57.5 (56.3, 58.7)		85.0	
Christian	47.7 (45.6, 49.9)		86.8	
Place of residence				
Rural	45.2 (44.78, 45.7)	*p* < 0.001	85.3	*p* < 0.001
Urban	63.3 (62.6, 64.0)		86.2	

^a^Wealth index, ^b^Body mass index, Underweight (BMI ≤ 18.4 kg/m^2^), Normal (18.5 kg/m^2^ ≤ BMI ≤ 24.9 kg/m^2^), Overweight (25.0 kg/m^2^ ≤ BMI ≤ 29.9 kg/m^2^), Obese (BMI ≥ 30 kg/m^2^).

Individuals who reside in urban areas (63.3%) had a significantly higher prevalence than rural areas (45.2%), however, a slight difference was observed in mean QoL. A similar pattern was also observed in the MPCE quintile, where the prevalence of multimorbidity in the poorest class was 39.8%, and a substantial difference was shifted to the richest class (62.6%). The prevalence of multimorbidity among individuals who did not have schooling is 44.1%, and those who had 10 + years of schooling is 61.2%. While their mean QoL scores are 84.1 and 88.5, respectively. An insignificant mean QoL score gap is observed among categories of caste and religion. An interesting pattern was observed among those individuals who had poor childhood health. Individuals with poor childhood health were more prone to have multimorbidity (57.3%) with a mean QoL score of 80.1 compared to their counterparts (50.6%) with a mean QoL score of 85.9. Furthermore, individuals who engaged in daily physical activity were less prone to develop multimorbidity and had a higher prevalence of good QoL at a later age. The widest differential for multimorbidity prevalence was observed between the underweight (39.5%) and obese (71.2%).

### 3.3 SEM

**Figure 3:**
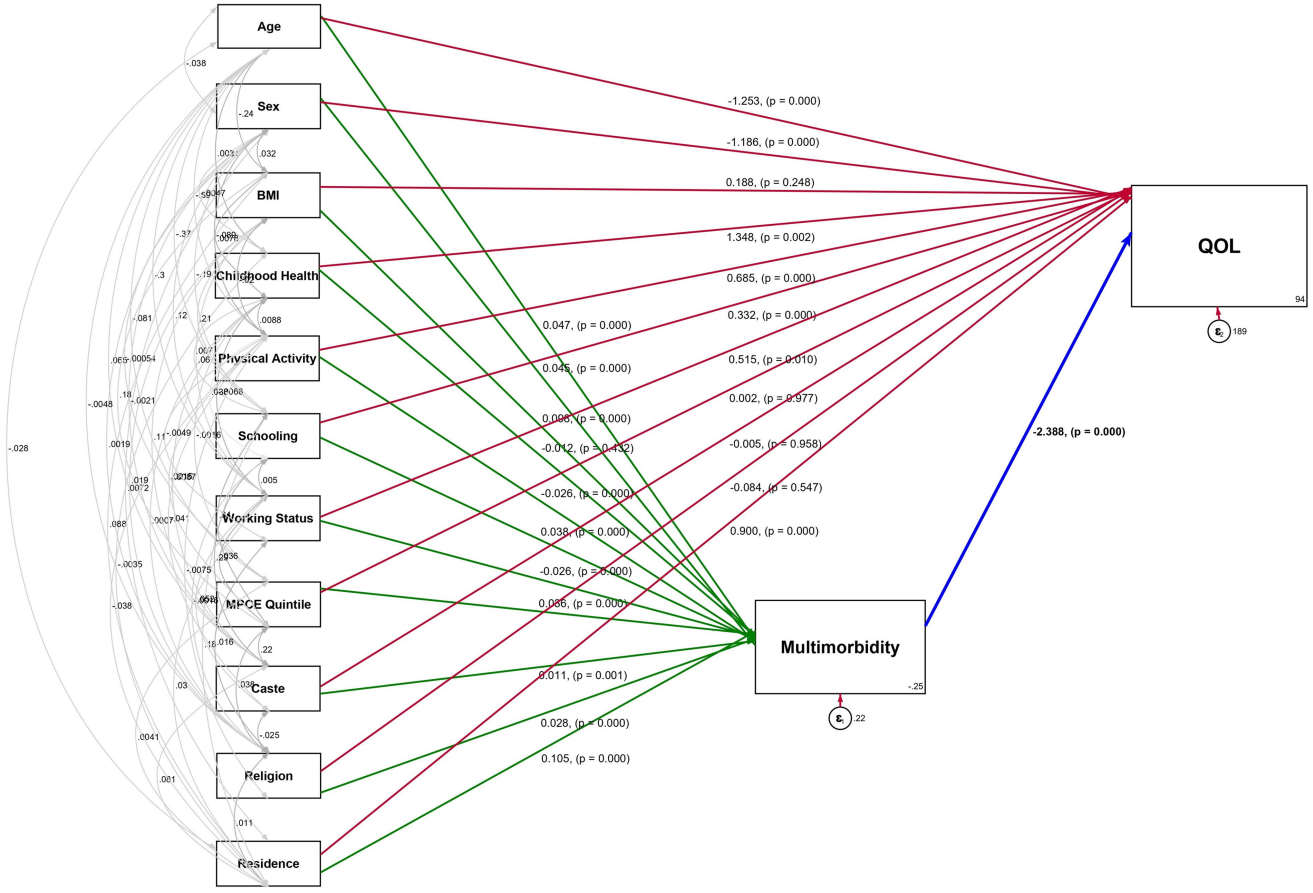
Path diagram of the Structural Equation Model (SEM) showing effect estimates on Quality of Life (QoL) and multimorbidity, LASI-WAVE 1 (2017–18), India.

Figure 3 illustrates the path diagram of the SEM, and Tables 3, 4 presents the direct, indirect, and total effects of exogenous factors on QoL, mediated by multimorbidity. The results indicated that multimorbidity was the leading significant predictor of QoL among older adults in India and had a notable negative effect (*β* = −2.39, *p* < 0.001), indicating that a sequential rise in multimorbidity led to poor QoL in the ageing population. Moreover, among all exogenous factors, the age and sex of the respondent showed the highest direct and indirect negative effect on QoL, suggesting that aged individuals and the female population had a higher likelihood of experiencing poor QoL. On the contrary, physical activity emerged as one of the robust and compelling positive contributors to QoL, having a significant direct effect (*β* = 0.69, *p* < 0.001) and a small but positive indirect effect through reduced multimorbidity (*β* = 0.06, *p* < 0.001).

**Table 3:** Direct effect of exogenous factors on multimorbidity, LASI-Wave 1 (2017–18), India.

Exogenous factors	Direct effect *β*(CI)
Age	0.05*** [0.04, 0.05]
Sex	0.05*** [0.03, 0.06]
BMI^a^	0.10*** [0.08, 0.11]
Childhood health	−0.01 [−0.04, 0.02]
Physical activity	−0.03*** [−0.04, −0.01]
Working status	−0.03*** [−0.04, −0.01]
Education level	0.04*** [0.03, 0.05]
MPCE quintile^b^	0.04*** [0.03, 0.06]
Caste	0.01** [0.00, 0.02]
Religion	0.03*** [0.02, 0.04]
Place of residence	0.10*** [0.08, 0.12]

*p < 0.05, **p < 0.01, ***p < 0.001; *β* = Beta Coefficient. ^a^Wealth index, ^a^Body mass index, Underweight (BMI ≤ 18.4 kg/m^2^), Normal (18.5 kg/m^2^ ≤ BMI ≤ 24.9 kg/m^2^), Overweight (25.0 kg/m^2^ ≤ BMI ≤ 29.9 kg/m^2^), Obese (BMI ≥ 30 kg/m^2^). ^b^Wealth index.

In addition, childhood health had a significant direct effect on QoL (*β* = 1.35, *p* < 0.001) but depicted no indirect effect, inferring that good childhood health leads to better QoL in later years. In contrast, BMI, MPCE quintile, caste, and religion had no significant direct effect on QoL but showed a significant indirect effect with negative association with QoL.

Our findings further revealed that higher educational attainment exerted a positive direct impact on QoL (*β* = 0.33, *p* < 0.001), implying that higher education levels correlate with better QoL. Although the indirect effect mediated by multimorbidity is negative (*β* = −0.09, *p* < 0.001), and indicates that the health advantages of education were partially diminished by the increased risk of multimorbidity among the educated older population. The aggregate effect remained favorable (*β* = 0.24, *p* < 0.01). Nonetheless, place of residence exhibited a varied effect, where urban residents had a strong positive direct effect on QoL (*β* = 0.9, *p* < 0.001). However, their indirect effect was negative (*β* = −0.25, *p* < 0.001), which led to an overall effect of *β* = 0.65 (*p* < 0.01). Moreover, factors such as caste and religion, exhibited a notable indirect effect but lack a significant direct impact on QoL. The final SEM fit statistics indicated an acceptable model fit, and total model fit indices were good with $X^{2}$ /df = 1.63 (*p* < 0.001), ARS = 0.112 (*p* < 0.001), CFI = 0.965 and RMSEA = 0.053 (Table 4).

**Table 4:** SEM with beta coefficient, direct, indirect, and total effects of background variables on QoL among older adults’ population, mediated by multimorbidity, LASI-Wave 1 (2017–18), India.

Exogenous factors	Direct effect *β*(95% CI)	Indirect effect *β*(95% CI)	Total effect *β*(95% CI)
Multimorbidity	−2.39*** [−2.81, −1.97]	No path	−2.39*** [−2.81, −1.97]
Age	−1.25*** [−1.38, −1.12]	−0.11*** [−0.14, −0.09]	−1.37*** [−1.5, −1.23]
Sex	−1.19*** [−1.64, −0.73]	−0.11*** [−0.16, −0.06]	−1.29*** [−1.74, −0.85]
BMI^a^	0.19 [−0.13, 0.51]	−0.23*** [−0.29, −0.18]	−0.05 [−0.36, 0.27]
Childhood health	1.35** [0.5, 2.19]	0.03 [−0.04, 0.1]	1.38*** [0.54, 2.21]
Physical activity	0.69*** [0.49, 0.88]	0.06*** [0.03, 0.09]	0.75*** [0.55, 0.94]
Working status	0.52* [0.13, 0.9]	0.06*** [0.03, 0.09]	0.58*** [0.19, 0.96]
Education level	0.33*** [0.17, 0.5]	−0.09*** [−0.12, −0.06]	0.24** [0.07, 0.41]
MPCE quintile^b^	0.01 [−0.16, 0.16]	−0.09*** [−0.11, −0.07]	−0.08 [−0.24, 0.07]
Caste	−0.01 [−0.2, 0.19]	−0.03** [−0.04, −0.01]	−0.03 [−0.23, 0.16]
Religion	−0.08 [−0.36, 0.19]	−0.07*** [−0.1, −0.03]	−0.15 [−0.42, 0.12]
Place of residence	0.9*** [0.47, 1.33]	−0.25*** [−0.31, −0.19]	0.65** [0.21, 1.09]

*p < 0.05, **p < 0.01, ***p < 0.001; *β* = Beta Coefficient. ^a^Wealth index, ^a^Body mass index, Underweight (BMI ≤ 18.4 kg/m^2^), Normal (18.5 kg/m^2^ ≤ BMI ≤ 24.9 kg/m^2^), Overweight (25.0 kg/m^2^ ≤ BMI ≤ 29.9 kg/m^2^), Obese (BMI ≥ 30 kg/m^2^). ^b^Wealth index.

## 4 Discussion

To the best of our knowledge, this is the first nationally representative study that extensively examines the dynamic effects of multimorbidity on the QoL among the older population in India. This study not only analyzed the direct effect of multimorbidity on QoL but also investigated the role of multimorbidity as an intermediating risk factor for exogenous factors on QoL. The first key findings of our study showed that hypertension imposes the highest burden on individuals, and this pattern persists across all age groups. Similarly, according to the WHO 2014 report, individuals aged 50 and above have a higher risk of hypertension diagnosis, especially in low and middle-income countries. In recent decades, rapid growth toward ageing and urbanization has served as the primary factors contributing to this heightened burden of multimorbidity (26–28). This huge burden of hypertension is also due to low awareness, late treatment, and control, particularly in lower socioeconomic groups (27). After hypertension, our research revealed that multimorbidity represents the second-highest age-specific burden affecting older Indian adults, with a sequentially increasing prevalence in the later age groups.

Considering the wide range of morbidities in this study, we estimated that half of the population is suffering from multimorbidity (51.4%), and this estimate was found to be consistent with other prior studies (23, 25, 29, 30). The “India: Health of the Nations’s State” reported that the occurrence of two or more morbidities reflects the epidemiological transition in India, where non-communicable diseases started to dominate over other diseases (31). Another key finding of this study showed a linear decline in the QoL over advancing age and a pronounced decrease in the QoL gradient was observed after 75 years of age. QoL score was constructed by considering its multidimensional nature. Physical health, psychological wellbeing, and social relationships, which were dimensions of QoL, were adversely affected by age (32, 33). Multiple studies have demonstrated that a significant decline in QoL was observed after the age of 75, attributed to reduced mobility, cognitive decline, increased dependency on others, cumulative health adversities, and depressive symptoms, which collectively contribute to diminished QoL among older adults (34, 35).

Additionally, our research suggested that individuals who had poor childhood health were linked to a higher burden of multimorbidity and a lower mean QoL score. Numerous studies have demonstrated that adverse health conditions during childhood, including malnutrition and higher susceptibility to infectious diseases, significantly impact health outcomes later in life (7, 20, 36). Chronic malnutrition, particularly stunting, hinders physical growth and cognitive development, leading to long-term deficits in functional capacity (37–41). Furthermore, micronutrient deficiencies compromise the immune system and increase the susceptibility to chronic illnesses. Additionally, obesity or being overweight leads to a heightened risk of developing NCDs such as diabetes, cardiovascular disorders, and hypertension (41–44). Hence, the compound effect of poor health adversities in childhood leads to a higher burden of morbidity and a diminished QoL later in life.

Our findings align with existing research that emphasizes the influence of socioeconomic factors on multimorbidity and QoL (4, 13). The findings of this study revealed a paradoxical relationship between education, QoL, and multimorbidity. While individuals with higher educational attainment report better QoL, they simultaneously face an elevated risk of multimorbidity, and households with higher wealth quintiles also bear a higher burden of multimorbidity. In contrast, the rural population exhibits a lower prevalence of multimorbidity but an equivalent mean QoL score compared to their urban counterparts. It is evident from previous studies that a higher level of schooling and higher wealth status generally reported better QoL due to enhanced health literacy, proactive health behaviors, and better utilization of healthcare, leading to a higher diagnosis rate (32). However, these demographic groups also have a higher prevalence of sedentary occupations, less physically active lifestyles, and dietary patterns that include the consumption of processed food, which also contribute significantly to the development of NCDs (45, 46). Moreover, the rural population often follows agrarian lifestyles with traditional diets and higher levels of physical activity, leading to a lower burden of multimorbidity and maintaining an equal QoL as the urban population. In contrast, there are shreds of evidence suggesting that the rural population suffers more from multimorbidity and poor QoL (12, 47). Another set of studies also observed that higher wealth groups exhibited a lower multimorbidity prevalence, attributed to the better and more effective preventive healthcare system and health-promoting environments (9, 22). However, these studies were often conducted in different settings than India or in a specific target group, which often compromised the generalizability of the result.

Furthermore, our study supports the existing literature by demonstrating that individuals with multimorbidity exhibit a 2.4 percent reduction in their QoL. Fortin et al. (2007) and Marengoni et al. (2011), have consistently shown that multimorbidity significantly diminishes QoL across older populations (1, 3). These studies underscore how the burden of multiple chronic conditions exacerbates limitations in daily functioning, increases healthcare utilization, and heightens psychological distress, ultimately reducing QoL. Other than multimorbidity, age has the highest impact on the QoL. Numerous studies highlight that increasing age often corresponds to declining QoL due to chronic conditions, physical limitations, and reduced social participation. The contextual nature of ageing—where cultural, healthcare and economic factors influence QoL (18, 32, 48). Gaspar et al. (2017) also argue that ageing alone is sufficient to predict QoL, as psychological resilience and social support can buffer the adverse effects of health conditions (32). Further, the identification of multimorbidity as a significant mediator linking socioeconomic factors to poor quality of life (QoL) emphasizes the need for targeted interventions addressing NCDs in India’s ageing population. The study’s observation that multimorbidity significantly deteriorates QoL (with a marked impact after age 75) signals an urgent requirement for improved geriatric healthcare services that focus on early detection, preventive care, and chronic disease management. These priorities align with India’s commitment to achieving SDG 3.4 by implementing cost-effective interventions to reduce NCD-related morbidity and mortality.

The SEM results showed that multimorbidity has significantly compounded the effect of socioeconomic and lifestyle factors on the QoL. BMI, wealth, caste, and religion did not have any direct effect on QoL. However, when multimorbidity functioned as a mediating factor, these factors demonstrated a statistically significant impact on QoL. The mediating role of multimorbidity aligns with theories suggesting that chronic illnesses amplify existing disparities (19, 20, 23). Barnett et al. (49) argued that multimorbidity disproportionately burdens socioeconomically disadvantaged populations, particularly in low-middle income settings such as India, where healthcare inequities are a critical concern. Furthermore, higher BMI is a well-known risk factor for chronic conditions such as diabetes, hypertension, and CVDs (46). These conditions collectively contribute to physical limitations, decreased healthcare utilization, and psychosocial stress, all of which reduce QoL. This pathway is especially pronounced in ageing populations, where physiological resilience to chronic conditions diminishes with age (3, 7, 36).

Another paradoxical finding of the study indicated that place of residence has the highest indirect impact on the QoL. Place of residence has a positive impact on QoL directly, but the mediating role of multimorbidity shifted the positive impact into a negative effect on the QoL. This duality aligns with the findings of Flies et al. (2019), who noted that the health benefits of urban living are often offset by environmental and lifestyle factors that contribute to chronic diseases. In contrast, rural settings showed disadvantages in terms of healthcare access and socioeconomic opportunities, might have lower rates of multimorbidity due to physical activity and traditional diets (50, 51). Thus, the mediating role of multimorbidity complicates the relationship between place of residence and QoL. This insight directly informs SDG 3.8, highlighting the need to strengthen India’s primary healthcare system to ensure comprehensive and affordable chronic disease care.

Furthermore, while wealth education and living in an urban area are traditionally considered protective, evidence suggests that higher socioeconomic groups are more prone to early diagnosis and detection of chronic conditions due to better healthcare access (52, 53). Urban and wealthier population are better positioned to access healthcare, often face lifestyle risks such as sedentary behavior, stress, and dietary changes, contributing to multimorbidity. This pattern underscores the need for inclusive healthcare policies that address both disadvantaged and seemingly advantaged populations to achieve the SDG 10. Therefore, preventive healthcare interventions must extend beyond vulnerable groups to include affluent and educated populations. Policies should integrate routine screenings, targeted health literacy programs, and lifestyle interventions customized for diverse socioeconomic backgrounds. To advance SDG 10, policymakers should implement tiered healthcare models that combine universal health coverage (SDG 3.8) with preventive strategies tailored to specific risk profiles. Strengthening primary healthcare systems to ensure equitable detection and management of chronic conditions is essential. Additionally, expanding community-based programs that emphasize behavioral changes, mental health support, and chronic disease prevention can mitigate multimorbidity risks. Addressing healthcare inequalities through such integrated approaches will reduce disparities and improve wellbeing for ageing populations in India.

## 5 Conclusion

In conclusion, this study underscores the critical interplay between multimorbidity and QoL among older adults in India, highlighting the significant mediating effects of multimorbidity of various socioeconomic health determinants on QoL. While factors such as age, physical activity, childhood health, BMI, and education emerged as important drivers of QoL, the wealth quintile presented an unexpected effect of direct or indirect associations, challenging conventional assumptions about economic status and health outcomes. Incorporating multimorbidity as a leading risk factor in parallel to conventional socioeconomic and demographic factors could enhance public health programs and resource allocation, and improve older adults’ QoL. Moreover, the integration of these findings with scalable community-based interventions has the potential to mitigate health disparities and enhance the wellbeing of the ageing population in India. This approach helps to achieve SDG 3, which aims to ensure healthy lives and promote wellbeing for all at all ages, as well as SDG 10, which seeks to reduce inequalities within and among countries. Future research should explore the paradoxical role of wealth, cultural, and environmental determinants in shaping QoL and the longitudinal impacts of multimorbidity.

The reported findings may suggest a hypothesis regarding the mediating effect of multimorbidity on poor quality of life (QoL), indicating that the age and sex of individuals played a significant role in both direct and indirect relationships to QoL. The revealed mediator is preventable at the population level by incorporating a dynamic approach at both modifiable risk factors and further deescalating the multimorbidity burden in the older adult population.

To address these findings, integrating sustainable healthcare strategies is crucial such as community-based health programs that promote healthy lifestyles, early screening, and chronic disease management can help mitigate the burden of multimorbidity. Additionally, leveraging telemedicine platforms can enhance healthcare accessibility in underserved regions, facilitating timely interventions and improved disease management. Policy reforms that prioritize integrated healthcare services, improved social support systems, and capacity building for healthcare providers are vital to ensuring that older adults receive comprehensive care.

## 6 Limitations of the study

The findings of the study provide a comprehensive and expanded perspective on multimorbidity and QoL, elucidating how multimorbidity influences the impact of additional risk factors on QoL. Nevertheless, this study is subject to certain limitations.

1. Although the structural equation model is a novel and powerful methodology for this study, we are unable to identify the causal relationship among study variables as our data is cross-sectional, consisting of only one wave.
2. The results cannot be generalized to the population with all kinds of multimorbidity compositions. Further, this study only quantified the burden of multimorbidity. It did not address severity, duration, and specific interactions of morbidity.
3. Our study included a more comprehensive list of morbidities that has not been considered in prior studies of multimorbidity and QoL in India, yet it is not possible to include function limitation, Activities of daily living (ADL), and Instrumental activities of daily living (IADL) as these have a bidirectional relationship with multimorbidity
4. This study lacks comprehensive data on participants’ emotional health and mental wellbeing. Future research should aim to collect more extensive information on these aspects to provide a more complete understanding of QoL.
5. QoL measurement variables in the LASI data rely on self-reporting, which is prone to bias. Hence, potential cultural biases not fully addressed.

## Data Availability

Publicly available datasets were analyzed in this study. This data can be found at: https://www.iipsindia.ac.in/lasi.

## Footnotes

^1^ www.R-project.org
