# Supplementary material for: Ageing, multimorbidity, and quality of life: a mediation analysis using longitudinal ageing study in India
Source: Front Public Health. 2025 Apr 25;13:1562479. doi: 10.3389/fpubh.2025.1562479 (PMC12061729; doi:10.3389/fpubh.2025.1562479)
Supplement: Supplementary file 1 [file Data_Sheet_1.pdf]

S1 Table: Detailed descriptions of all the exogenous factors, Longitudinal Ageing Study in India (LASI), wave-1, 2017–2018

Exogenous factors	Descriptions
Sex	Sex of the individual
Age Group	Current age group of the individual
Residence	Place of current residence of the individual
Childhood Health	In general, what was the overall childhood health of individual up to age 16 years.
MPCE quintile	MPCE - Monthly per capita consumption expenditure quintiles
BMI	Body mass index as Underweight (BMI≤ 18.4 kg/m2), Normal (18.5 kg/m2 ≤ BMI ≤ 24.9 kg/m2), Overweight (25.0 kg/m2 ≤ BMI ≤ 29.9 kg/m2), Obese (BMI ≥ 30 kg/m2)
Highest level of Schooling	Highest level of education that individual completed
Caste Category	SC - Scheduled Castes<br>ST - Scheduled Tribes<br>OBC - Other Backward Classes
Working Status	Working status of the individual
Religion	Religion of individual
Physical Activity	The type of physical activity included in the individual's lifestyle.

S2 Table: Kaiser-Meyer-Olkin Measure of Sampling Adequacy

KMO	0.796

S1 Figure: Scree plot of PCA presenting the factors retain from all the variables/items

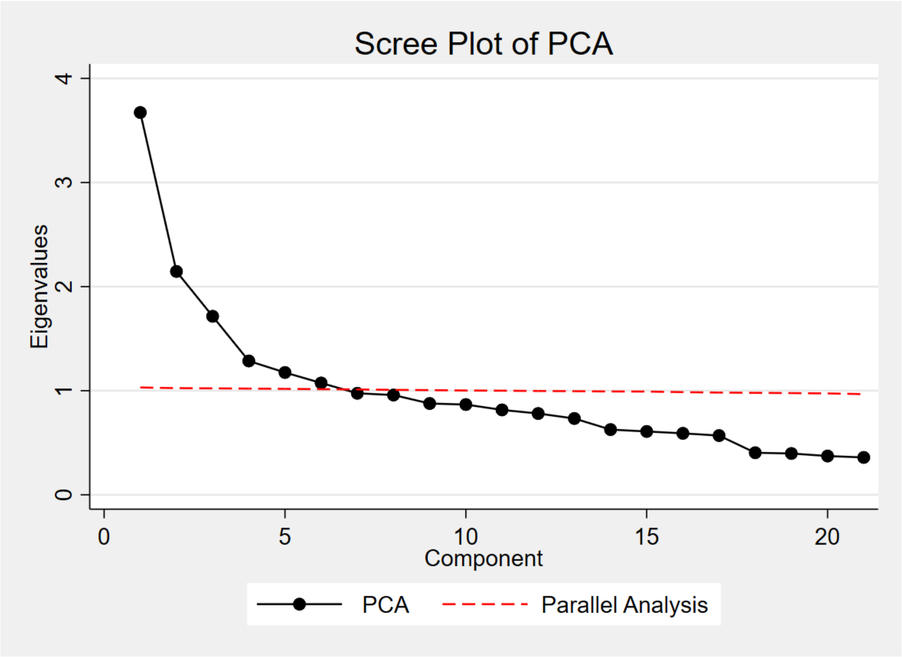

S3 Table: Variance explained by the retained factors

Factor	Variance	Difference	Proportion	Cumulative
Factor 1	3.44026	1.57316	0.1638	0.1638
Factor 2	1.8671	0.22647	0.0889	0.2527
Factor 3	1.64063	0.1439	0.0781	0.3309
Factor 4	1.49673	0.04637	0.0713	0.4021
Factor 5	1.45037	0.27818	0.0691	0.4712
Factor 6	1.17218	.	0.0558	0.527

S4 Table: Factors loadings of all the factors from PCA

**Variable**	**Factor 1**	**Factor 2**	**Factor 3**	**Factor 4**	**Factor 5**	**Factor 6**	**Uniqueness**
Pain	0.1498	0.3605	-0.0531	0.1775	-0.268	-0.3688	0.6055
Life satisfaction	0.0619	0.1845	0.0628	0.3421	0.5538	-0.0811	0.5279
Alone	0.0276	-0.0986	-0.0322	-0.0182	0.4371	-0.4384	0.6049
Friend	0.0856	-0.0051	0.082	0.0843	0.2002	0.4479	0.7381
Satisfied	0.0583	0.1308	0.0533	0.19	0.6943	-0.0031	0.4584
Safe	0.0245	0.1415	0.0876	-0.1974	0.478	0.0378	0.7029
Finance	-0.0085	0.0165	-0.0285	0.1068	-0.0893	0.7098	0.4756
Concentrated	0.1191	0.5708	0.0629	-0.1894	0.1891	0.1344	0.5663
Spiritual	0.0116	-0.0071	0.8905	0.0769	0.0131	-0.0028	0.2007
Satisfied	-0.0027	-0.0834	0.1159	0.7796	0.0287	0.0457	0.3689
Depressed	0.0685	0.7201	0.0197	0.0353	0.1588	0.0592	0.4463
Happy	0.0364	0.0227	0.146	0.7435	0.1563	0.049	0.3972
Peace	0.0081	0.0681	0.8811	0.091	0.0435	0.0076	0.2088
Low energy	-0.0394	-0.7163	-0.0591	0.0832	-0.0299	0.0405	0.4724
Sleep	-0.1232	-0.4983	-0.0366	-0.1632	0.0858	0.2482	0.6396
Toilet	0.6897	0.1507	-0.0134	0.0698	-0.0797	-0.1508	0.4675
Bedding	0.741	0.1165	0.0024	0.0578	-0.067	-0.1036	0.4188
Eating	0.6901	0.0345	-0.0065	0.0026	0.0493	-0.0018	0.52
Walk	0.7965	0.0121	0.015	-0.0178	0.0694	0.0538	0.3572
Bath	0.8219	0.0033	0.0098	-0.0026	0.0385	0.028	0.3221
Dress	0.7441	0.0198	0.0317	-0.0161	0.0785	0.0698	0.4336

S5 Table: Cronbach alpha and average interitem correlation for the validly of the QoL construct

**Item**	**Average Interitem correlation**	**Cronbach alpha**
Life satisfaction	0.096	0.68
Living arrangement	0.104	0.70
Have friend	0.103	0.70
Satisfied living arrangement	0.097	0.68
Feel safe	0.101	0.69
Financial support receives	0.105	0.70
Trouble with concentration	0.098	0.68
Spiritual	0.099	0.69
Satisfied	0.103	0.70
Depressed	0.096	0.68
Happy	0.099	0.69
Peace	0.098	0.69
Energy	0.098	0.68
Trouble with sleep	0.097	0.68
Trouble with toilet	0.090	0.66
Trouble with bedding	0.089	0.66
Trouble with eating	0.091	0.67
Trouble with walk	0.089	0.66
Trouble with bath	0.088	0.66
Trouble with dress	0.090	0.66
Trouble with Pain	0.100	0.69
**Test scale**	**0.097**	**0.69**
